# Detection of Skeletal Lesions by Whole Body Multidetector Computed Tomography in Multiple Myeloma has no Impact on Long-Term Outcomes Post Autologous Hematopoietic Cell Transplantation

**DOI:** 10.4021/wjon551w

**Published:** 2012-08-26

**Authors:** Baldeep Wirk, Charles H. Bush, Wei Hou, Leslie Pettiford, Jan S. Moreb

**Affiliations:** a Department of Medicine, College of Medicine, University of Florida, USA; b Department of Radiology, College of Medicine, University of Florida, USA; c Department of Biostatistics, College of Medicine, University of Florida, USA

**Keywords:** Whole-body CT, Multiple myeloma, Bone disease

## Abstract

**Background:**

Multiple myeloma (MM), a plasma cell malignancy, is the most common cancer to involve the skeleton. Skeletal related events such as pathologic fractures and lytic bone lesions have been associated with poor prognosis. Whole body multidetector computed tomography (WBCT) has been shown to be the most sensitive imaging modality in detecting small osteolytic lesions (< 5 mm) in the spine. The significance of lytic lesions detected only by CT is unknown as is their impact on overall survival of MM. The aim of this study was to evaluate the impact of lytic bone lesions seen only by WBCT on progression free survival (PFS) and overall survival (OS) in MM patients after hematopoietic cell transplantation (HCT).

**Methods:**

We evaluated 72 patients who had WBCT and conventional radiographic skeletal survey (CSS) after initial or salvage chemotherapy prior to HCT.

**Results:**

Forty-one patients (57%) had more findings on WBCT than CSS, 31 patients (43%) had no differences in the two imaging techniques, 9 patients had no bone lesions on either modality, and 5 patients had lesions only identified by WBCT and not on CSS. PFS and OS were similar in patients with lesions seen by CSS irrespective of whether additional lesions were noted by WBCT; similarly, in patients without lesions on CSS, OS and PFS were better than patients with lytic lesions, but detection of occult lesions by WBCT did not adversely affect PFS or OS.

**Conclusions:**

Our study shows that although WBCT is more sensitive in defining existing myelomatous bony disease in MM, these additional findings may not have any impact on PFS and OS of MM patients. Only patients without any bone lesions on conventional skeletal survey had significantly better PFS and OS. This suggests CSS remains the gold standard for evaluating myeloma bone disease.

## Introduction

Multiple myeloma (MM), a clonal plasma cell malignancy, is the second most common hematologic malignancy and the most common cancer to involve the skeleton [1, 2], 90% of patients will develop lytic bone lesions in the course of their disease with 80% presenting at diagnosis with lytic bone lesions detected by conventional radiographic skeletal survey (CSS) [3], 15% of MM patients present with osteopenia and osteoporosis [3]. These lesions cause significant morbidity with vertebral compression fractures and pathologic fractures in 60% of patients as well as hypercalcemia [3, 4]. Skeletal related events such as pathologic fractures and lytic bone lesions have been associated with poor prognosis [5].

Lytic bone lesions as detected by CSS are used as criteria for diagnosis of symptomatic MM and for staging. The Durie-Salmon (DS) staging system was introduced in 1975 and is based on serum levels of hemoglobin, calcium and monoclonal protein as well as the presence or absence of lytic bone lesions as assessed by CSS [6]. The DS staging system has been found to correlate with patient survival and tumor mass [6]. The International Staging System (ISS) introduced in 2005 is based on the serum β-2 microglobulin and albumin [7]. The International Myeloma Working Group (IMWG) includes hypercalcemia, renal insufficiency, anemia and lytic bone lesions as assessed by plain radiographs (CRAB) in the related organ or tissue impairment (ROTI) to diagnose symptomatic MM and as an indication for therapy [8, 9]. This is based on studies showing at least one lytic lesion on CSS in MM patients is associated with a median time to progression of 10 months [9].

IMWG consensus guidelines recommend CSS as the gold standard for evaluating myeloma bone disease; however spinal and pelvic magnetic resonance imaging (MRI) can be used to give complementary information to plain radiography and is recommended in all patients with solitary plasmacytoma [10]. Either MRI or computed tomography (CT) of the spine is the procedure of choice to assess spinal cord compression [10]. Recently the whole body multidetector computed tomography (WBCT) has been shown to be more sensitive in detecting small osteolytic lesions of the spine (< 5 mm) as compared to whole body MRI and 18F-fluorodeoxyglucose positron emission tomography (FDG-PET) [11, 12]. This is due to the higher z axis resolution of the high quality real time 3-dimensional images and multiplanar images of WBCT which can better evaluate the extent of osteolytic bone lesions [11]. The significance of lytic lesions detected only by CT is unknown as is their impact on overall survival. It is also unknown whether lytic lesions detected only by CT can be included in the definition of symptomatic MM and as an indication for therapy.

In our institution, both WBCT and CSS have been used to evaluate MM bone disease during the pre transplant evaluation. The aim of this study was to assess the differences between these two imaging modalities and to evaluate the impact of lytic bone lesions seen only by WBCT on progression free survival (PFS) and overall survival (OS) after hematopoietic cell transplantation (HCT) for multiple myeloma.

## Materials and Methods

A retrospective study of MM patients referred to the University of Florida, Bone Marrow Transplant program for transplant evaluation was conducted to identify patients who had both CSS and WBCT. Between January 2005 and December 2008, we identified 72 consecutive MM patients treated by initial hematopoietic peripheral blood stem cell transplantation who had had both CSS and WBCT during their pre transplant evaluation, within 30 days of the first autologous HCT. The aim of this study was to assess the differences between these two imaging techniques in the detection of myelomatous bone disease and to evaluate the impact of these differences on PFS and OS after HCT. The study was approved by the institutional review board at the University of Florida. A musculoskeletal radiologist with over 25 years experience reviewed each WBCT and CSS.

### Conventional skeletal survey

A complete radiographic skeletal survey was performed according to the IMWG guidelines: a posteroanterior view of the chest; anteroposterior and lateral views of the cervical spine (including open mouth view), thoracic spine, lumbar spine, humeri and femora bilaterally; anteroposterior and lateral views of the skull; and an anteroposterior view of the pelvis [10]. Our institutional protocol also included anteroposterior radiography of the forearms and calves bilaterally. The total imaging time was 30 minutes. Myelomatous bone disease was defined as: 1) lytic “punched out” lesions on radiography; 2) endosteal scalloping or discrete small lytic lesions (< 1 cm); 3) mottled, demineralized areas without discrete lesions.

### Whole body multidetector computed tomography

All patients were examined supine with a Siemens Sensation 16 or Toshiba Aquilon 64 helical multidetector CT scanner. The area scanned was from the vertex of the skull through the feet with 5 mm section thickness with additional 2 mm sections reconstructed at 1 mm intervals through the vertebral column and reformatted in sagittal and curved coronal planes to look for compression fractures. A typical scan technique used a tube voltage of 135 kV with variable mAs. The mean scan time was less than 2 minutes. The mean effective dose was 52 mSv. No oral or intravenous contrast was used. Images were reconstructed in both soft tissue and bone detail algorithm, and were viewed on picture archiving and communication system (PACS) workstations.

Myelomatous bone disease on CT was defined as: 1) Large, lytic “punched out” or expansile bony lesions; 2.) discrete small lytic lesions (< 1 cm); 3) A diffusely permeative pattern of rarefaction in the axial skeleton, sometimes appearing as diffuse osteopenia; 4) Pathologic fracture; 5) Discrete, homogeneous soft tissue attenuation intramedullary lesions (with or without endosteal scalloping) in the proximal appendicular skeleton that could not be attributed to hematopoietic bone marrow.

### Statistical analysis

For the purposes of this study, patients were divided into the following groups: Group 1: Patients who had more myelomatous bone disease detected on WBCT than CSS; Group 2: Patients without any differences between CSS and WBCT in the detection of myelomatous bone disease; Group 3: Patients without any myelomatous bone disease detected by either modality; Group 4: Patients with myelomatous bone lesions detected by WBCT but not by CSS; Group 5: patients in groups 3 and 4 together.

Comparisons were carried out using the chi-square test for categorical variables and the non-parametric Mann-Whitney test for the continuous variables. The Cox proportional hazard model was used to conduct univariate and multivariate survival analyses. A P value of .05 or less was considered statistically significant. The patients in each group 1 through 5 were compared for PFS and OS using the Kaplan-Meier method. The survival curves were compared using the log rank test. Patient and disease characteristics were collected as outlined in Table 1. OS was defined as the time elapsed from the first autologous HCT until death from any cause or at the last time of contact. PFS was defined as the time between first autologous HCT and first disease relapse, progression or last contact.

**Table 1:** Patient and Disease Characteristics

Characteristics	Group 1 (n = 41)	Group 2 (n = 31)
Male/female	22/19	17/14
Age, median (range) years	58 (25 - 75)	59 (43 - 75)
Durie-Salmon/ ISS		
I A/I	2/19	3/11
II A/I	12/11	9/11
III A/IIIB/III	23/4/5	13/6/5
B2M, mg/L, median (range)	2.27 (0.84 - 14.1)	2.6 (1.45 - 69.6)
Median follow-up (range), months	36 (6 - 84)	31 (11 - 73)
Lines of therapy before HCT		
1	28	20
2	9	7
3	4	4
Conditioning for first autologous HCT		
Melphalan	32	18
Busulfan/cyclophosphamide +/- etoposide	9	13
Cytogenetics/FISH		
Normal	25	24
Complex/hyperdiploid and del 13 by cytogenetics	4	1
Hyperdiploid without deletions	6	3
FISH + chromosomes 13,14, or 17	3	3
Del Y	2	1
Time to first auto HCT: median (range) months	7 (3 - 60)	7 (3 - 39)
Tandem autologous/autologous HCT	5	4
Tandem autologous/allogeneic HCT	5	6
Salvage autologous/autologous HCT	4	1
Salvage autologous/allogeneic HCT	6	2
Maintenance lenalidomide	4	1
Maintenance thalidomide/interferon	5	6

Abbreviations: HCT: hematopoietic cell transplant; FISH: fluorescence in situ hybridization; ISS: international staging system; B2M: beta 2 microglobulin; WBCT: whole body computerized tomography; CSS: conventional skeletal survey; Group 1: Patients who had more myelomatous bone disease detected on WBCT than CSS; Group 2: Patients without any differences between CSS and WBCT in the detection of myelomatous bone disease.

## Results

**Figure 1:**
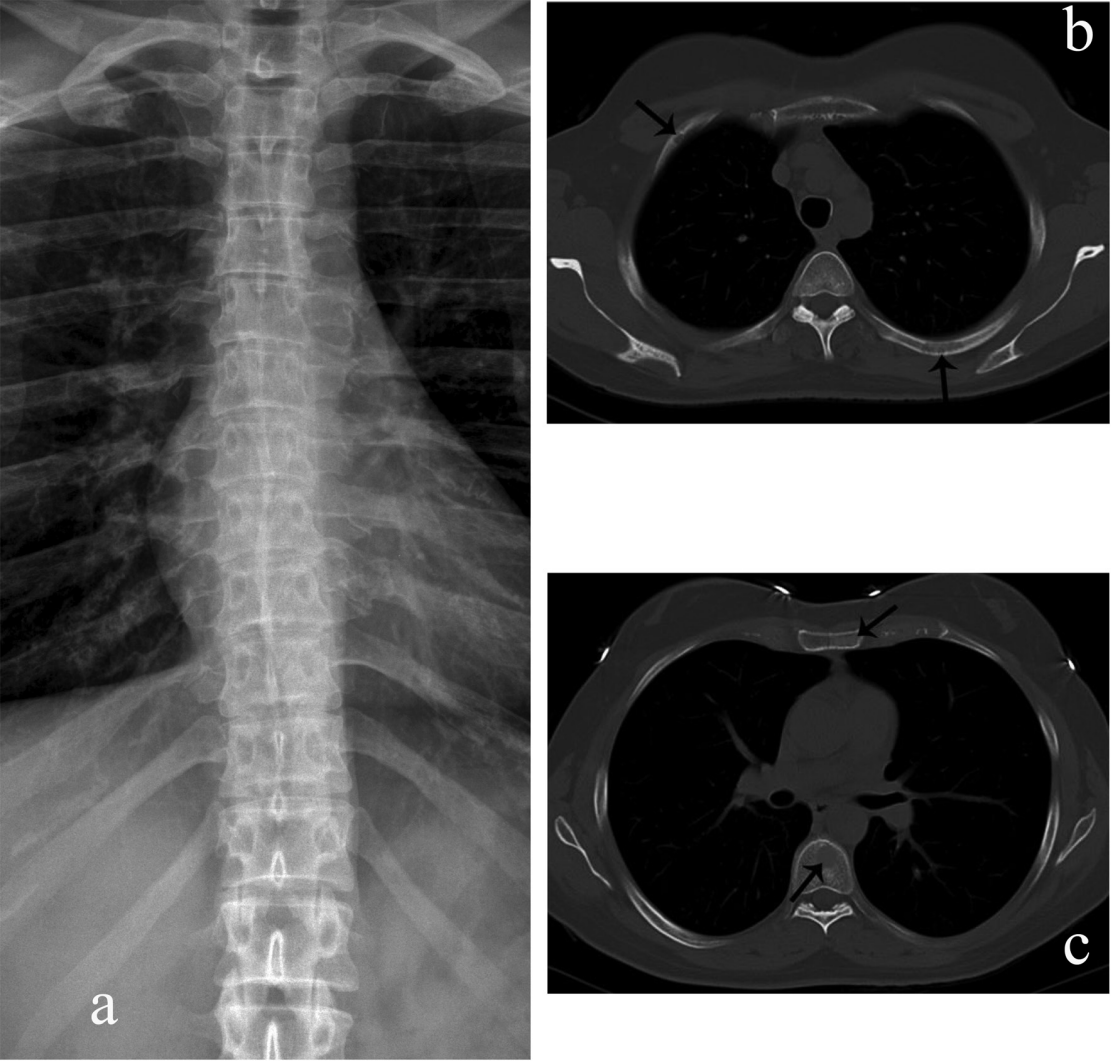
Rib, sternal, and vertebral body myelomatous lesions seen by WBCT (arrows) (b, c) but not conventional skeletal radiography (a).

**Figure 2:**
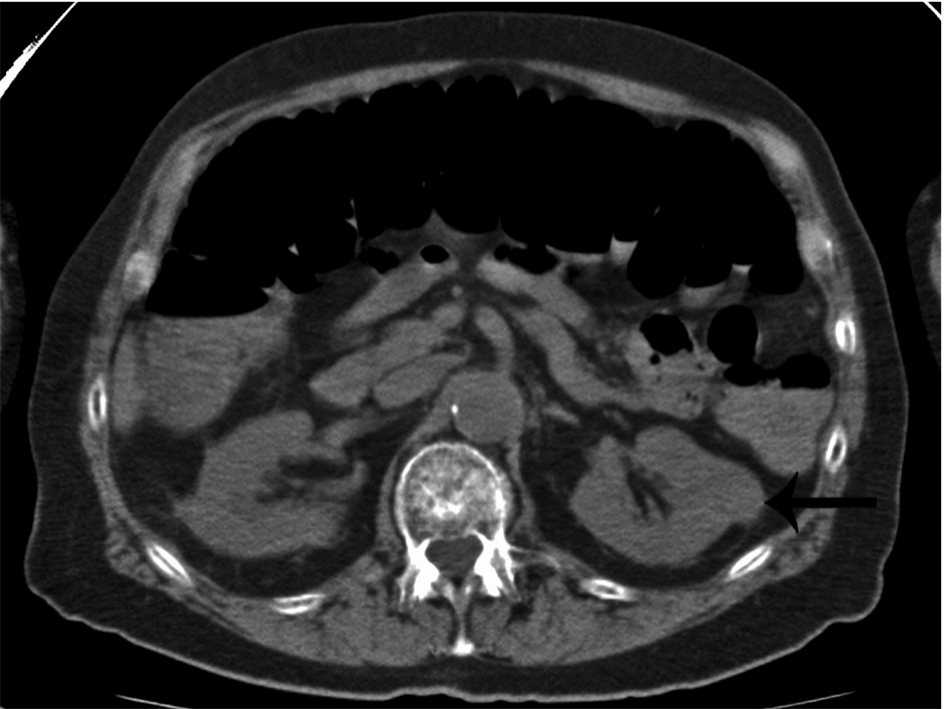
Incidental finding of a non-cystic, exophytic left renal mass in a patient with multiple myeloma (arrow). This mass was later found to be a renal cell carcinoma.

The 72 MM patients who had both CSS and WBCT performed within 30 days of the first autologous HCT are listed in Table 1 with their disease characteristics and treatment details. Forty one patients (57%) were in group 1 and had more findings on WBCT than CSS; 31 patients (43%) were in group 2 and had no differences in the two imaging techniques; 9 of these patients were in group 3 and had no bone lesions on either modality; and 5 patients (group 4) had lesions only identified by WBCT and not on CSS. Group 5 consisted of 14 patients (19%) who had a normal CSS and 5 of these patients had bone lesions detected by WBCT alone. Examples are shown in Figure 1. Two patients had incidental renal masses identified which were surgically resected and found to be renal cell carcinoma (one example is shown in Fig. 2). Table 1 lists the patients in groups 1 and 2 who had tandem autologous or allogeneic HCT as well as salvage autologous or allogeneic HCT and/or maintenance therapy and they are similarly distributed between the two groups. Table 2 lists the patient and disease characteristics of Group 5 (negative CSS) compared to all other patients. No statistically significant difference was found between Group 5 and all the other patients including stage of MM, time from diagnosis to transplant, lines of chemotherapy prior to transplant or adverse cytogenetics. Having a negative CSS was the only significant factor on multivariate analysis for PFS and this was also maintained on multivariate analysis for OS (Table 3, 4). Additional significant factors on multivariate analysis for OS were DS stage IIIB and ISS II.

**Table 2:** Patient and Disease Characteristics of Group 5 (Negative Conventional Skeletal Survey) Compared to all Other Groups

Characteristic	Group 5 (n = 14)	All Other Patients (n = 58)
Male/female	5/9	34/24
Age, median (range) years	59 (47 - 75)	56 (35 - 75)
Durie-Salmon stage		
I A	1	4
II A	7	14
III A/IIIB	3/3	33/7
International staging system		
I	3	26
II	6	16
III	3	7
missing	2	9
Lines of therapy before HCT		
1	10	38
2	3	13
3	1	7
Cytogenetics/FISH		
Normal	11	38
Complex/hyperdiploid and del 13 by cytogenetics	0	5
Hyperdiploid without deletions	1	8
FISH + chromosomes 13,14, or 17	2	4
Del Y	0	3
Time to first autologous HCT: median (range) months	6 (4 - 24)	7 (3 - 60)
Tandem autologous/autologous HCT	2	7
Tandem autologous/allogeneic HCT	5	6
Salvage autologous/autologous HCT	0	5
Salvage autologous/allogeneic HCT	0	8
Maintenance lenalidomide	0	5
Maintenance thalidomide/interferon	2	9

Abbreviations: HCT: hematopoietic cell transplant; FISH: fluorescence in situ hybridization; ISS: international staging system.

**Table 3:** Risk Factor Analysis for Progression Free Survival

Variable	Univariate			Multivariate		
	Hazard ratio	95% CI	P value	Hazard ratio	95% CI	P value
Disease stage DS						
IIA vs IA	0.884	0.292 - 2.675	0.8274	1.166	0.342 - 3.972	0.8058
IIIA vs IA	1.337	0.465 - 3.845	0.2898	1.722	0.575 - 5.157	0.3311
IIIB vs IA	1.439	0.420 - 4.930	0.3350	4.219	0.994 - 17.902	0.0509
Disease stage ISS						
II vs I	1.467	0.800 - 2.692	0.2156	1.41	0.699 - 2.845	0.3376
III vs I	0.742	0.304 - 1.808	0.5110	0.533	0.189 - 1.508	0.2358
Cytogenetics/FISH						
3 vs 1	1.178	0.496 - 2.797	0.7107	0.997	0.391 - 2.44	0.9598
2 vs 1	1.750	0.483 - 6.254	0.3894	2.411	0.638 - 9.109	0.1943
Negative vs Positive conventional skeletal survey	0.522	0.253 - 1.077	0.0784	0.396	0.172 - 0.910	0.0291
Gender						
Male vs Female	0.998	0.577 - 1.726	0.9944	1.088	0.603 - 1.963	0.7795
Age	1.005	0.977 - 1.033	0.7452	1.005	0.973 - 1.039	0.7534

Abbreviations: DS: Durie-Salmon stage; ISS: International staging system; FISH: fluorescence in situ hybridization; Definitions of cytogenetics: 1-poor prognosis, complex abnormalities with del 13, 17p, or t(4:14), or any of these abnormalities; 2- Intermediate prognosis, normal chromosomes, Del 13 by FISH only; 3- Good prognosis, hyperdiploidy, t(11,14).

**Table 4:** Risk Factor Analysis for Overall Survival

Variable	Univariate			Multivariate		
	Hazard ratio	95% CI	P value	Hazard ratio	95% CI	P value
Disease stage DS						
IIA vs IA	0.754	0.083 - 6.876	0.8026	1.27	0.111 - 14.485	0.8472
IIIA vs IA	2.116	0.275 - 16.264	0.4715	4.57	0.534 - 39.147	0.1655
IIIB vs IA	3.839	0.461 - 31.947	0.2133	11.972	1.196 - 116.23	0.0346
Disease stage ISS						
II vs I	7.70	2.765 - 21.837	0.0001	7.234	2.179 - 24.015	0.0012
III vs I	4.579	1.321 - 15.872	0.0164	3.426	0.809 - 14.516	0.0946
Cytogenetics/FISH						
3 vs 1	1.044	0.308 - 3.539	0.9452	0.26	0.058 - 1.154	0.0763
2 vs 1	1.089	0.181 - 6.54	0.9257	0.232	0.031 - 1.724	0.1532
Negative vs Positive conventional skeletal survey	0.136	0.018 - 1.01	0.051	0.061	0.007 - 0.554	0.013
Gender Male vs Female	0.803	0.358 - 1.798	0.593	0.875	0.37 - 2.069	0.761
Age	1.03	0.987 - 1.074	0.1809	1.047	0.987 - 1.111	0.126

Abbreviations: DS: Durie-Salmon stage; ISS: International staging system; FISH: fluorescence in situ hybridization; Definitions of cytogenetics: 1- poor prognosis,complex abnormalities with del 13, 17p, or t(4:14), or any of these abnormalities; 2- Intermediate prognosis, normal chromosomes, Del 13 by FISH only; 3- good prognosis, hyperdiploidy, t(11,14).

**Figure 3:**
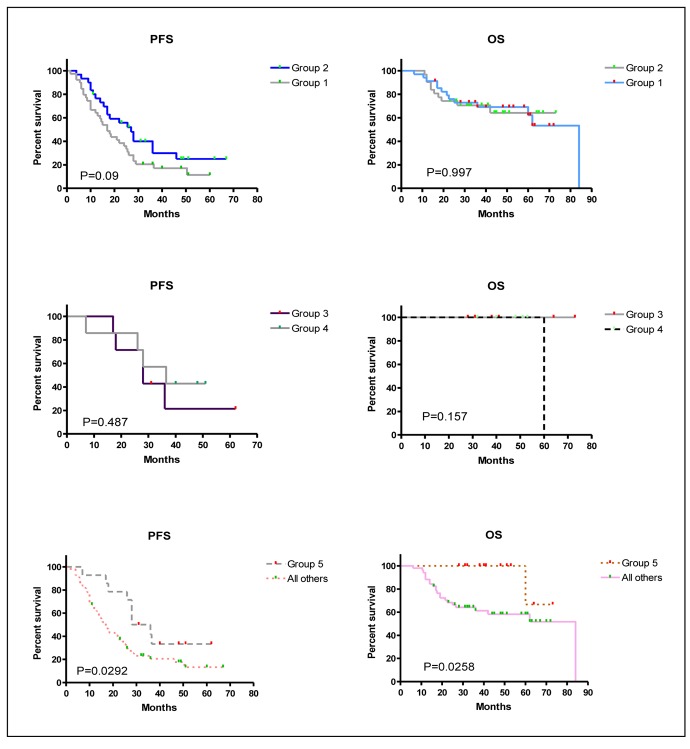
Kaplan-Meier overall survival (OS) and progression-free survival (PFS) curves for the different groups of patients. P values are displayed within the figure for each comparison. Group 1: Patients who had more myelomatous bone disease detected on WBCT than CSS. Group 2: Patients without any differences between CSS and WBCT in the detection of myelomatous bone diseaseGroup 3: Patients without any myelomatous bone disease detected by either modality. Group 4: Patients with myelomatous bone lesions detected by WBCT but not by CSS.Group 5: patients in groups 3 and 4 together.

The impact of the added myelomatous bone disease findings detected by WBCT on PFS and OS were analyzed by comparing groups 1 versus 2, groups 3 versus 4, and group 5 versus all other groups (Table 5 and Fig. 3). WBCT detected more myelomatous bone disease in 57% of patients than CSS but this had no impact on PFS or OS (Fig. 3, top panels). Nine patients had no myelomatous bone disease on the CSS or WBCT and their PFS and OS was the same as those who had bony lesions identified by WBCT alone (Fig. 3, middle panels). It was only in the subgroup analysis of the 14 patients who had negative CSS (group 5) who had significantly better PFS and OS in comparison to all other patients who had bone disease detected by CSS (Fig. 3, bottom panels).

**Table 5:** Comparison of Survival Curves Using the Logrank Test

Groups of patients	Median PFS (months)	Median OS (months)	Hazard ratio	95% confidence interval	P value
Groups 1 vs. 2	17 vs. 27	84 vs. not yet reached	PFS: 0.627 OS 1.001	PFS: 0.36 - 1.1 OS: 0.43 - 2.34	PFS: 0.09 OS: 0.997
Groups 3 vs. 4	28 vs. 36.5	Not yet reached vs. 60	PFS: 1.544 OS: 0.00	PFS: 0.41 - 6.66 OS: 0.0008 - 3.18	PFS: 0.487 OS: 0.157
Groups 5 vs. all others	32 vs. 17	Not yet reached vs. 84	PFS: 0.47 OS: 0.145	PFS: 0.28 - 0.93 OS: 0.13 - 0.87	PFS: 0.0292 OS: 0.0258

Abbreviations: Group 1: Patients who had more myelomatous bone disease detected on WBCT than CSS; Group 2: Patients without any differences between CSS and WBCT in the detection of myelomatous bone disease; Group 3: Patients without any myelomatous bone disease detected by either modality; Group 4: Patients with myelomatous bone lesions detected by WBCT but not by CSS; Group 5: patients in groups 3 and 4 together.

## Discussion

Recently there is a greater use of modern cross sectional imaging to define myelomatous bone disease, with some transplant centers using WBCT rather than CSS. The number of lytic bone lesions detected by CSS was found to be an independent predictor of survival in the DS staging system [6]. The significance of lytic lesions detected only by WBCT is unknown as is their impact on overall survival. Our study shows that although WBCT is more sensitive in defining existing myelomatous bony disease in MM, these additional findings may not have any impact on PFS or OS of MM patients after autologous HCT. Furthermore, it was only in the subgroup analysis of patients without any bone lesions on CSS that had significantly better PFS and OS when compared to all other patients. No statistically significant difference was found between Group 5 (negative CSS) and all the other patients with respect to stage of MM, time from diagnosis to transplant, lines of chemotherapy prior to transplant or adverse cytogenetics. Despite being retrospective, this study is unique in its uniform application of WBCT and CSS in MM patients undergoing initial autologous HCT and addresses the clinical implications of these additional findings.

The new International Staging System for MM is based on the serum β-2 microglobulin and albumin levels [7]. The IMWG guidelines stipulate presence of damage, specifically ROTI (hypercalcemia, renal insufficiency, anemia and lytic bony lesions on CSS) as being symptomatic MM and an indication for therapy [8]. Our study also suggests that indications for therapy should not only be based on lytic lesions detected by WBCT alone, but rather a combination of clinical findings, conventional skeletal radiography and laboratory values.

The Durie-Salmon PLUS staging system is based on the number of bony lytic lesions detected by modern cross-sectional imaging (by WBCT-PET or MRI of the spine and pelvis) integrated with the original DS staging system [13]. The concordance of the DS and the Durie-Salmon PLUS staging systems was examined in a study using whole body MRI versus conventional skeletal radiography to stage untreated MM patients [14], 14% of patients were upstaged and 41% were downstaged but in a prediction of overall survival, the Durie-Salmon PLUS staging system was not better than the DS staging system. The overall survival was 33.6 months versus 33.5 months versus 31.6 months whether the patients were staged the same, down staged or upstaged and this was not statistically significant [14]. This is in contrast to a study of MM patients with osteoporosis measured by lumbar spine quantitative computed tomography who died an average of 18 months earlier than those without osteoporosis but only 11% of patients had HCT in that study [15].

The prognostic implications of lytic lesions in MM are controversial. The bone marrow microenvironment is so altered at sites of bone destruction that new bone formation is rare despite a response to therapy [16]. Even patients responding to chemotherapy or in complete remission can have skeletal disease progression due mainly to increased osteoclast activity and suppressed osteoblast function, which accounts for the lack of healing of bony lesions [3, 17]. Healing of myelomatous bone disease has been seen in case reports of bortezomib, a proteasome inhibitor with important regulation of osteoblast differentiation, and this can happen irrespective of response to bortezomib [18-20]. In our study, WBCT and CSS were not done at diagnosis but upon referral to our transplant program within 30 days of the first autologous HCT. This was at a median of 7 months from diagnosis of MM. However, due to the pathogenesis of myelomatous bone lesions with lack of bone healing due to absence of new bone formation, the results are an accurate reflection of the extent of myelomatous bone disease [3, 16, 17].

Whole body multidetector computed tomography (WBCT) has been shown to be more sensitive in detecting small osteolytic lesions (< 5 mm) in the spine as compared to whole body MRI and 18F-fluorodeoxyglucose positron emission tomography (FDG-PET) [11, 12]. WBCT can detect early small lytic bone lesions in the vertebral bodies, scapulae, ribs and sternum (Fig. 1) and determine the fracture risk better than CSS [21, 22]. In addition, over 30% of trabecular bone loss is needed before lytic bone lesions can be detected by plain films [21, 22]. This is significant because the most common areas of myelomatous bone disease are in the vertebrae, ribs, skull and shoulders [23]. WBCT is faster without a need for repositioning frail patients with bone pain; however, the cost of the WBCT is substantially more than CSS (at our institution WBCT costs $2,822 US dollars in comparison to $607 US dollars for CSS). The slightly higher radiation doses of the WBCT are not usually an issue in a disease that mainly affects the elderly. WBCT is nonspecific for osteopenia or osteoporosis. Although there is a risk of renal insufficiency if intravenous iodinated contrast is used in patients with MM, especially those with Bence-Jones proteinuria, this is not an issue in standard noncontrast WBCT. Also in our study, 2 patients were diagnosed with renal cell carcinoma after the finding of incidental renal masses on WBCT (Fig. 2).

In conclusion, detection of bony lytic lesions by modern cross sectional imaging with WBCT alone may not have any impact on PFS or OS of MM patients undergoing HCT. Perhaps this is because the prognostic impact of myelomatous bony disease can only be fully defined by further understanding of the pathogenesis of the enhanced osteoclast activity and osteoblast dysfunction that characterizes multiple myeloma. Furthermore, our study suggests CSS should remain the gold standard radiologic method for all myeloma patients.
